# Predicting Treatment Nonresponse in Hispanic/Latino Children Receiving Silver Diamine Fluoride for Caries Arrest: A Pilot Study Using Machine Learning

**DOI:** 10.3389/froh.2021.695759

**Published:** 2021-07-26

**Authors:** Ryan Richard Ruff, Bidisha Paul, Maria A. Sierra, Fangxi Xu, Xin Li, Yasmi O. Crystal, Deepak Saxena

**Affiliations:** ^1^Department of Epidemiology and Health Promotion, New York University College of Dentistry, New York, NY, United States; ^2^Department of Molecular Pathobiology, New York, NY, United States

**Keywords:** silver diamine fluoride, dental caries-most common childhood diseases, machine learning, treatment nonresponse, microbiome

## Abstract

**Objectives:** Silver diamine fluoride (SDF) is a nonsurgical therapy for the arrest and prevention of dental caries with demonstrated clinical efficacy. Approximately 20% of children receiving SDF fail to respond to treatment. The objective of this study was to develop a predictive model of treatment non-response using machine learning.

**Methods:** An observational pilot study (*N* = 20) consisting of children with and without active decay and who did and did not respond to silver diamine fluoride provided salivary samples and plaque from infected and contralateral sites. 16S rRNA genes from samples were amplified and sequenced on an Illumina Miseq and analyzed using QIIME. The association between operational taxonomic units and treatment non-response was assessed using lasso regression and artificial neural networks.

**Results:** Bivariate group comparisons of bacterial abundance indicate a number of genera were significantly different between non-responders and those who responded to SDF therapy. No differences were found between non-responders and caries-active subjects. Prevotella pallens and Veillonella denticariosi were retained in full lasso models and combined with clinical variables in a six-input multilayer perceptron.

**Discussion:** The acidogenic and acid-tolerant nature of retained bacterial species may overcome the antimicrobial effects of SDF. Further research to validate the model in larger external samples is needed.

## 1. Introduction

Dental caries, a polymicrobial disease caused by a diverse bacterial ecosystem where acid produced by acidogenic and aciduric bacteria in direct contact with tooth surfaces erodes the enamel and dentin of the tooth [1], is the most prevalent childhood disease in the world [2, 3]. Untreated dental caries affects more than 20% of children in the United States and over 50% of children have ever experienced caries, higher amongst low-income and minority children [4–6], populations typically characterized by lower dental service utilization [7]. To address this unmet need, school-based caries prevention programs can supplement traditional care, increasing access to dental services and reducing oral health inequities [8, 9].

Silver diamine fluoride (SDF) is a liquid therapeutic agent that combines the antibacterial effects of silver and the re-mineralizing effects of fluoride [10] with proven clinical efficacy in arresting ~80% of caries lesions in enamel and dentin [11]. The use of SDF for arresting cavitated caries lesions in primary teeth is recommended by the American Association of Pediatric Dentists and the American Dental Association [12] with growing utilization in pediatric dental programs across the United States.

The mechanisms associated with refractory growth of caries initially treated using SDF remain unclear. In pragmatic studies, this phenomenon of non-response to the therapeutic effect of SDF has been observed in children with and without prior caries experience, in those living in both fluoridated and non-fluoridated communities, and was not related to sex or age [13]. The presence of plaque in lesions resistant to the caries arresting action of SDF suggests that the microbiome composition of the individual oral cavity and/or the specific caries site may be a strong determinant in the preventive action of SDF [11, 14]. Silver ions have demonstrated an antimicrobial effect against specific bacteria and the development of new biofilms [15, 16]. However, most studies with cariogenic bacteria have been conducted in *in-vitro* biofilm models with one or more species while the human biofilm is composed of multitude of species, the interaction of which may influence the effect of SDF in preventing caries.

We conducted a pilot study to explore the innate commonalities between the microbiomes of children that may increase susceptibility or resistance to caries and impact the effectiveness of SDF therapy. Study objectives included determining whether (1) caries prone or resistant children harbor significantly different oral microbiota; (2) microbiomes are more or less resistant to preventive agents; and (3) these data can be used along with clinical indicators to predict treatment non-response. Results for objectives (1) and (2) were previously reported [17]. In this paper, we present preliminary results supporting objective (3). Our null hypothesis was that there was no association between bacterial abundance, clinical indicators, and treatment non-response.

## 2. Methods

This study received approval from the New York University School of Medicine Institutional Review Board (#s19-00692, “The role of the oral microbiome in predicting disparities in caries and responsiveness to caries prevention: An observational pilot study,” 23 June 2019) and is reported according to the STROBE checklist.

### 2.1. Design and Participants

This was an observational pilot study conducted from June to November 2019. Twenty children aged 5–13 years were recruited from the New York University College of Dentistry dental clinic at the time of their routine visit. Healthy subjects were enrolled if they had untreated dental caries (*N* = 5), were caries free (*N* = 5), were scheduled to receive silver diamine fluoride during their current visit (*N* = 5), or had previously received SDF and presented with re-occurrence of caries (*N* = 5). Children in each group were unique and did not crossover. Excluded subjects were those who had antibiotic therapy in the previous 2 months or those who could not follow the instructions to collect the saliva samples. All study participants were of Hispanic/Latino origin. Each subject provided parental informed consent and child assent.

### 2.2. Silver Diamine Fluoride Application

SDF application was performed by a licensed dentist (YC) and followed standard clinical practice guidelines published by the American Association of Pediatric Dentistry [18] using a single application of 38% SDF solution consisting of 25% silver, 8% ammonia, 5% fluoride, and 62% water for one minute.

### 2.3. Sample Collection

Untreated dental caries or re-occurrence of caries was determined by subjects presenting with visible cavitated lesions corresponding to an International Caries Detection and Assessment System (ICDAS) score of 3 or higher. Patients received silver diamine fluoride if they presented with visual cavitated lesions consisting of an ICDAS score of 4 or higher. Biological samples were collected once from each of the 20 study participants in a single visit. All subjects received a visual-tactile oral examination and provided unstimulated saliva. Specimen collection followed established methods for oral microbiome studies [19]. For children with caries, plaque samples were taken from caries sites and from the buccal surface of the second primary molar on the opposite side of the treated or untreated cavity. Children without caries provided supragingival plaque samples from the buccal surface of the second maxillary molar on both sides of the mouth. Supragingival plaque was collected with a single stroke using a sterile Gracey mini curette. TE buffer was used to collect the samples. Samples were placed in separate pre-barcoded micro centrifuge tubes with transport buffer and placed in a portable freezer. All biological samples were collected by a licensed dentist. Collected samples were then transported to the study laboratory (transit time <5 min) and immediately stored at −80°C for further analyses.

Sociodemographic and other clinical data for participants were obtained using pre-study questionnaires or were extracted from electronic health records. Data included subject age, sex, ethnicity, the number of hours since the last meal was consumed and since the last time the subject brushed his or her teeth, visible plaque index score, and caries history. While there was no control over the time of the last meal or their last brushing, this data were collected and both were at least 1 h in all subjects.

### 2.4. Microbiome Analysis

Bacterial genomic DNA was extracted using QIAamp PowerFecal kit (Qiagen). 8,057,899 Illumina-generated sequence reads were obtained from 60 samples (consisting of a single saliva sample and plaque samples from two sites from each study subject) and processed using QIIME 2. In total, 3,963 amplicon sequence variants (ASVs) were generated DNA purity was verified using NanoDrop 2000 spectrophotometer (Thermo scientific) and quantified fluorometrically using Quant-iT PicoGreen assay (Invitrogen). Each of the samples were further diluted or concentrated to 10 ng/ul. The variable V4 region of the bacterial rRNA gene was amplified in duplicates using barcoded forward and reverse primers 341F (5-CCTACGGGNGGCWGCAG-3) and 805R (5-GACTACHVGGGTATCTAATCC-3), each with overhang adapter sequences (IDT). PCR was done using 2x Kapa HiFi Hotstart ReadyMix DNA polymerase (KapaBiosystems). Reactions were verified by agarose-gel electrophoresis and amplicons were purified using AMPure XP beads. A second PCR was performed using dual indices from Illumina Nextera XT index kits (Illumina) followed by second purification with AMPure XP beads. The amplicons were further quantified by PicoGreen assay and diluted to 4 nm. The samples were then pooled together, denatured, and sequenced on Illumina MiSeq platform (paired end, 2*300 bp). Only results for sequenced saliva samples were used in analysis.

### 2.5. Data Analysis

Descriptive statistics (means, standard deviations, and proportions) were calculated for clinical and sociodemographic data. Any differences across study groups were assessed using χ^2^ tests for categorical variables and analysis of variance for continuous variables. *T*-tests with multiple comparison adjustments were used to detect any simple bivariate differences across the relative abundance of operational taxonomic units (OTUs). Adjusted models for the association between taxonomic units and treatment non-response was then assessed using lasso regression [20, 21]. Study groups were collapsed into those who were non-responders to SDF and all other subjects (caries active, caries free, and responders) due to small sample sizes in the pilot. All available OTUs were used as potential predictors with a single outcome consisting of two classes (non-responder vs. all other study participants). Analysis used k-fold cross-validation to produce values for λ, after which a logistic lasso was performed. Coefficients of retained variables were saved and plots were produced for coefficient change across λ values.

Retained OTUs and clinical indicators for time elapsed since prior meal and brushing, age, and visible plaque index (VPI) were used as input variables for an artificial neural network. The included OTUs were first normalized using the function *(x-min(x))/(max(x)-min(x))*. A multilayer perceptron consisting of five hidden nodes, six inputs, and one output was constructed and computed using backpropagation. Predictions were compared to observed values but due to the small sample size no test set was used for validation.

Analysis was conducted in R v3.6.3 (http://www.r-project.org) using the glmnet and neuralnet packages. Statistical significance was set at 0.05.

## 3. Results

The average age of study participants was 9.1 (SD = 2.15) with ~55% of the sample consisting of females (Table 1). Sex was unequally distributed across treatment groups (e.g., 20% female in caries active and 80% in SDF groups). The average elapsed time since the previous meal and the last toothbrushing was 4.35 (SD = 3.62) and 3.25 (SD = 1.97) hours, respectively. The average visible plaque index score across the study sample was 2 (SD = 0.56).

**Table 1:** Clinical and sociodemographic data (*N* = 20).

	**All**	**Caries active**	**Caries free**	**Non-responders**	**SDF**
**Variable**	**Mean (SD)**	**Mean (SD)**	**Mean (SD)**	**Mean (SD)**	**Mean (SD)**
Age (years)	9.1 (2.15)	9.4 (2.7)	9.2 (1.48)	9.8 (2.59)	8 (1.87)
Time last meal (h)	4.35 (3.62)	7 (4.69)	2.6 (1.67)	3.2 (2.17)	4.6 (4.28)
Time last brush (h)	3.25 (1.97)	3 (1.58)	2.8 (2.39)	4 (2.35)	3.2 (1.92)
Visible plaque index	2 (0.56)	2 (0.71)	1.8 (0.84)	2 (0)	2.2 (0.45)
Sex (females) %	55	20	60	60	80

**Figure 1:**
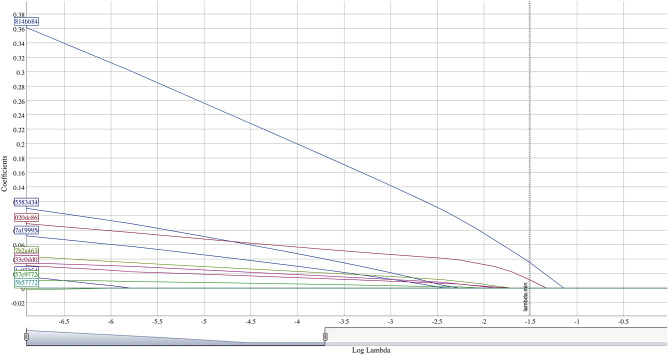
Coefficient path of OTUs at different values of lambda.

Bivariate group comparisons for abundance of specific genera in non-responders (Table 2) indicates that Oribacterium, Prevotella, Aggregatibacter, Fusobacterium, Peptostreptococcaceae, and Campylobacter were significantly different comparing non-responders to SDF-treated subjects. When compared to caries-free subjects, only Absconditabacteria and Peptostreptococcus were significantly different. There were no significant differences between non-responders and caries-active individuals. In full lasso models, two species were retained (Table 3) at the lambda minimum: Pallens (genus Prevotella) and Denticariosi (genus Veillonella). The coefficient path showing changes in estimates for OTUs for included lambdas is shown in Figure 1.

**Table 2:** Genera abundance comparisons for SDF, caries-free, and caries-active groups vs. non-response group (“NR”).

**Group**	**Taxa**	**Mean/SD**	**NR Mean/SD**	***p*-value**
SDF	Oribacterium	50.6 (60.98)	126.6 (37.32)	0.02695256
	Prevotella	1005.8 (360.65)	1503.8 (319.45)	0.02662165
	Aggregatibacter	207.6 (61.82)	79.2 (70.27)	0.02860014
	Fusobacterium	442.2 (98.44)	219.6 (117.16)	0.02969937
	Campylobacter	258.6 (42.29)	96.4 (101.13)	0.01704905
	Peptostreptococcaceae [XI G-9]	13.0 (8.97)	2.2 (4.4)	0.0385394
Caries free	Absconditabacteria [(SR1) G-1]	74.2 (48.01)	5.6 (11.2)	0.02393104
	Peptostreptococcus	82.4 (28.9)	35.8 (14.63)	0.03700165
Caries active	NA			

*Only significant comparisons are shown. No significant associations were found for the caries active vs. non-response group (“NA”)*.

**Table 3:** Retained and potentially relevant OTUs for treatment non-response with average abundance for those retained amongst non-responders.

	**Phylum**	**Family**	**Genus**	**Species**	**Mean (SD)**
**Retained**	Bacteroidetes	Prevotellaceae	Prevotella	Pallens	1503.8 (319.45)
	Firmicutes	Veillonellaceae	Veillonella	Denticariosi	3052.2 (855.67)
**Non-retained**	Bacteroidetes	Prevotellaceae	Prevotella	Multiformis	263.8 (21.17)
	Fusobacteria	Leptotrichiaceae	Leptotrichia	NA	278.6 (170.76)
	Actinobacteria	Micrococcaceae	Rothia	Dentocariosa	507.0 (263.92)

**Figure 2:**
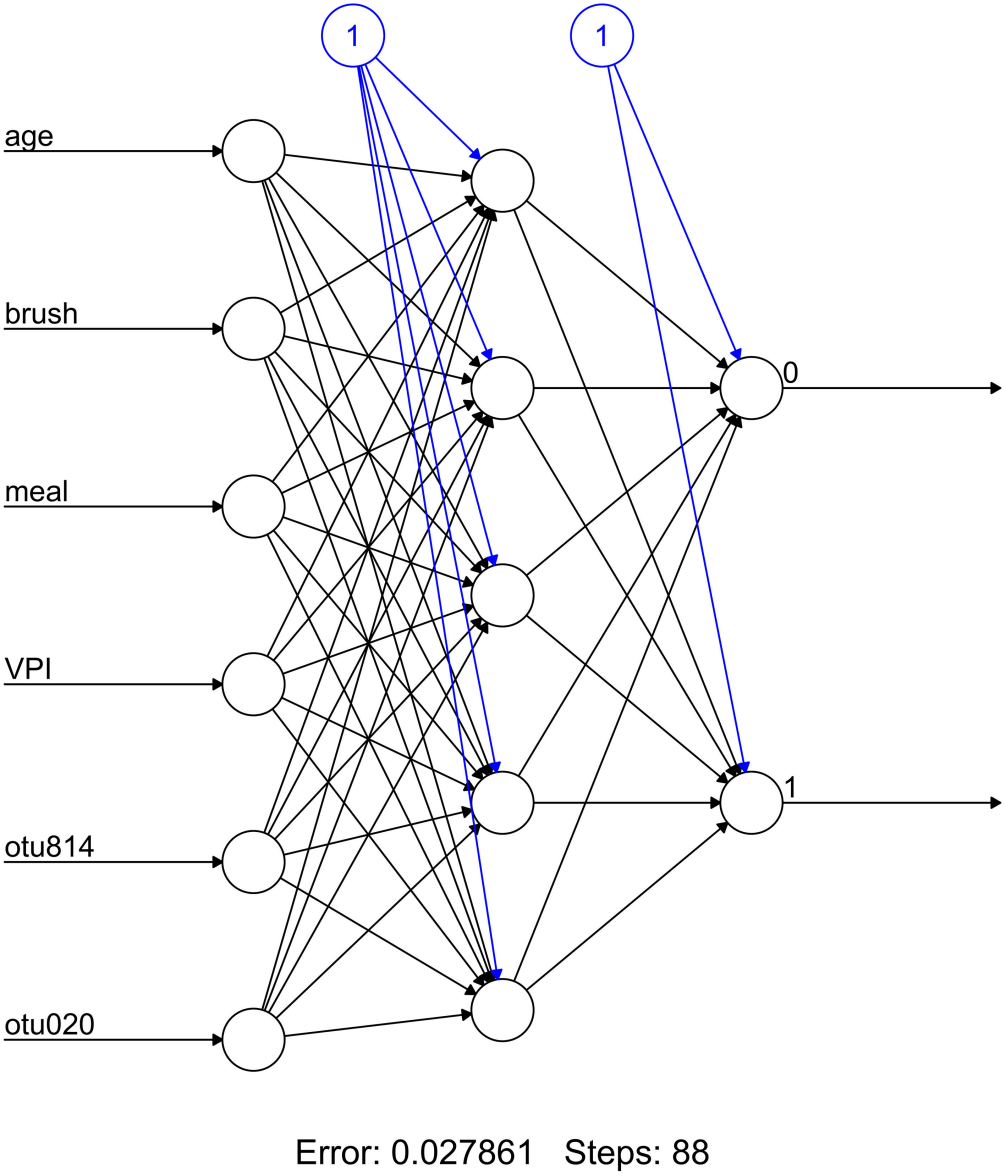
Artificial neural network topology for OTUs and clinical variables (weights not shown).

At smaller estimates of lambda, additional OTUs were present (species Multiformis, Dentocariosa of the Rothia genus, and the genus Leptotrichia). Given the small sample size, these OTUs may be relevant in future analyses. An interactive graph of the coefficient path is available in a public GitHub repository (www.github.com/ryanruff/nonresponse). The network topology map for the two retained species and clinical indicators is shown in Figure 2. Associated weights for node connections (black lines) and bias (blue lines) are available in the GitHub repository.

## 4. Discussion

Silver diamine fluoride combines the antibacterial behavior of silver with the remineralization properties of fluoride that creates an unfavorable environment for collagen degradation, and can inhibit the progression of dental caries [22]. We previously reported that there are distinct microbiota within plaque and saliva samples that can successfully differentiate between non-responders to SDF and other groups, though no differences were found in microbial composition between infected or contralateral sites [17]. In this study, our approach considered two methods to yield a predictive model for treatment non-response using a sample of patients who had previously received silver diamine fluoride for dental caries. While research using machine learning in oral epidemiology is limited, previous studies have successfully applied ML-approaches to predict malodor using salivary microbiota [23], hypertension from gingival inflammation [24], oral cancer [25], oral poliovirus vaccine immunogenicity [26], temporomandibular joint osteoarthritis, and radiographic detection of tooth and dental restorations [27]. Notably, a recent review concluded that artificial intelligence, particularly machine learning and deep learning, can use data from the oral microbiome to predict systemic disease [28].

The efficiency, clinical effectiveness, and cost-benefits of silver diamine fluoride make it an attractive option for the prevention and arrest of dental caries at the population level, specifically for those from low socio-economic backgrounds [29, 30]. Previous studies have shown that a non-trivial proportion of children with and without baseline disease continue to manifest new caries [13] following treatment with SDF. As a result, research that can support the successful identification of these non-responders to SDF therapy can lead to alternative intervention strategies, such as atraumatic restorations, glass ionomer sealants, or fluoride varnishes, and reduce oral health inequities. This research may also lead to a greater understanding of the potentially complex interaction between the mechanistic action of SDF, hygiene behavior, and diet.

Non-responders were compared to children with and without active caries, as well as those who have responded to the therapeutic effects of SDF. Bivariate results indicate no differences in bacterial abundance between those that fail to respond to SDF and those with untreated carious lesions, and the greater number of differences were restricted to the SDF-treated group. Results using lasso regression indicated that two bacteria, Prevotella pallens and Veillonella dentocariosa, may be useful in predicting treatment nonresponse. Prevotella is proteolytic, saccharolytic, and acid-tolerant [31], an overabundance of which has been associated with dental caries [32], in particular *P. pallens* [33]. The genus Veillonella consists of small strictly anaerobic gram-negative cocci that have been associated with severe early childhood caries [34]. Veillonella relies on lactate and other organic acid as its nutrient source and poorly adhere to host tissue. However, they are known to coaggregate with Streptococcus species such as *S. mutans* in biofilm formation in oral cavity [35]. These acidogenic and acid-tolerant bacterial species may overcome the antimicrobial effects of SDF, though the mechanistic action of this is still unknown.

Other species that were initially associated but were not retained at the lambda minimum included Prevotella multiformis, Rothia dentocariosa and the genus Leptotrichia. Prevotella multiformis, first isolated from oral gingival plaque, are gram-negative bacilli or cocci that produce acid from glucose, lactose, sucrose, glycerol, D-mannose, and D-raffinose [36]. Rothia dentocariosa is a gram-positive bacteria with a variable morphology ranging from coccoid to branched filamentous, generally grows in aerobic and microaerophilic conditions producing acid from glucose, sucrose, maltose and glycerol [37], and has been previously associated with periodontal inflammatory disease. Notably, several gram-positive bacteria produce lipoproteins that induce TLR-2 mediated inflammation. Thus, Rothia denticariosa may be acting in concert with other gram-positive bacteria to invoke host-immune response and regulate inflammatory effects in dental caries [38]. Bacteria belonging to genus Leptotrichia are non-motile facultative anaerobe gram-negative bacilli that are found in the oral cavity. Similar to streptococcus mutans, they ferment carbohydrates and produce organic acids such as lactic acid, and traces of acetic, formic and succinic acid. As *S. mutans* adheres to tooth surfaces and produces lactic acid causing demineralization of tooth enamel, Leptotrichia may us a similar mechanism to contribute to dental caries [39]. Further study on the potential of action of these bacteria in overcoming the effect of SDF is warranted.

The clinical definition of non-response to silver diamine fluoride is not yet fully explicated. In this study, any subject previously treated with SDF that presented with new incidence of decay was classified as a non-responder. However, an alternative classification might be to group individuals by the severity of non-response and the importance of individual operational taxonomic units might depend, in part, on the chosen clinical definition. Finally, independent variables for this study were limited solely to specific clinical indicators and OTUs produced by saliva and plaque samples taken from carious lesions and contralateral sites.

While the small sample size from this pilot study prohibited out of sample validation for testing purposes, our approach produced a restricted function for classifying whether a subject receiving silver diamine fluoride would appropriately respond to treatment using specific operational taxonomic units and clinical variables. Additionally, the age range of study subjects resulted in mixed dentition for our sample, which may exhibit differential response to the mechanistic action of SDF and the potential role of the oral microbiome. Further research should also consider effects in children from varying racial/ethnic backgrounds, as well as the confounding or mediating role of oral hygiene and dietary intake. Notably, the value of the coefficients from lasso models and neural networks are less important than the predictive power of the model and are thus unreported, though they can be extracted from Supplementary Files. As would be the case with any regularization approach, our results are only one of many possible models that could be useful in predicting non-response. Retained OTUs do not necessarily mean that those not included in the model are irrelevant. Regardless, this preliminary investigation has identified variables that are predictive of treatment non-response, which may be useful in making predictions in larger, independent datasets.

## Data Availability Statement

The raw data supporting the conclusions of this article will be made available by the authors, without undue reservation.

## Ethics Statement

The studies involving human participants were reviewed and approved by the New York University School of Medicine Institutional Review Board (#s19-00692, The role of the oral microbiome in predicting disparities in caries and responsiveness to caries prevention: An observational pilot study, 23 June 2019). Written informed consent to participate in this study was provided by the participants' legal guardian/next of kin.

## Author Contributions

RRR conceived and developed the study, performed statistical analysis, and wrote the manuscript. YOC contributed to study development, enrolled subjects, collected samples, and wrote the manuscript. BP, MAS, and FX analyzed study samples and wrote the manuscript. DS and XL contributed to study development and wrote the manuscript. All authors contributed equally to this manuscript.

## Conflict of Interest

The authors declare that the research was conducted in the absence of any commercial or financial relationships that could be construed as a potential conflict of interest.

## Publisher's Note

All claims expressed in this article are solely those of the authors and do not necessarily represent those of their affiliated organizations, or those of the publisher, the editors and the reviewers. Any product that may be evaluated in this article, or claim that may be made by its manufacturer, is not guaranteed or endorsed by the publisher.

## References

[1] Simón-Soro A Mira A. Solving the etiology of dental caries. Trends Microbiol. (2015) 23:76–82. 10.1016/j.tim.2014.10.010 25435135

[2] Marcenes W Kassebaum NJ Bernabé E Flaxman A Naghavi M Lopez A. Global burden of oral conditions in 1990-2010. J Dent Res. (2013) 92:592–7. 10.1177/0022034513490168 23720570 PMC4484374

[3] Kassebaum NJ Smith AGC Bernabé E Fleming TD Reynolds AE Vos T. Global, regional, and national prevalence, incidence, and disability-adjusted life years for oral conditions for 195 countries, 1990-2015: a systematic analysis for the global burden of diseases, injuries, and risk factors. J Dent Res. (2017) 96:380–7. 10.1177/0022034517693566 28792274 PMC5912207

[4] Dye BA Li X Thorton-Evans G. Oral health disparities as determined by selected Healthy People 2020 Oral Health objectives for the United States. NCHS Data Brief. (2009) 104:1–8. 23101968

[5] Dye BA Li X Thorton-Evans G. Dental caries and sealant prevalence in children and adolescents in the United States. NCHS Data Brief. (2011) 2015:1–8. 25932891

[6] Griffin SO Wei L Gooch BF Weno K Espinoza L. Vital signs: dental sealant use and untreated tooth decay among U.S. school-aged children. MMWR Morbid Mortal Week Rep. (2016) 65:1141–5. 10.15585/mmwr.mm6541e1 27764075

[7] Treadwell HM. The nation's oral health inequities: who cares. Am J Public Health. (2017) 107:5. 10.2105/AJPH.2017.303957 28661796 PMC5497895

[8] National Institute of Dental and Craniofacial Research. Department of Health and Human Services PHS, Centers for Disease Control and Prevention, and the National Institutes of Health, National Institute of Dental and Craniofacial Research. (2003).

[9] Dye BA Tan S Smith V Lewis BG Barker LK Thornton-Evans G. Trends in oral health status: United States, 1988-1994 and 1999-2004. Vital Health Stat. (1988) 11:1–92. 17633507

[10] Yasmi O Crystal RN. Evidence-based dentistry update on silver diamine fluoride. Dent Clin North Am. (2019) 63:45–68. 10.1016/j.cden.2018.08.011 30447792 PMC6500430

[11] Gao SS Zhao IS Hiraishi N Duangthip D Mei ML Lo ECM. Clinical trials of silver diamine fluoride in arresting caries among children. JDR Clin Transl Res. (2016) 1:201–10. 10.1177/2380084416661474 30931743

[12] Slayton RL Urquhart O Araujo MWB Pilcher L Banfield L Carrasco-Labra A. Evidence-based clinical practice guideline on nonrestorative treatments for carious lesions: a report from the American Dental Association. J Am Dent Assoc. (2018) 149:837–86. 10.1016/j.adaj.2018.07.002 30261951

[13] Richard Ruff R Saxena D Niederman R. School-based caries prevention and longitudinal trends in untreated decay: an updated analysis with Markov chains. BMC Research Notes. (2020) 13:25. 10.1186/s13104-020-4886-8 31924271 PMC6954604

[14] Fung MHT Duangthip D Wong MCM Lo ECM Chu CH. Randomized clinical trial of 12% and 38% silver diamine fluoride treatment. J Dent Res. (2018) 97:171–8. 10.1177/0022034517728496 28846469 PMC6429575

[15] Mei ML Chu CH Low KH Che CM Lo EC. Caries arresting effect of silver diamine fluoride on dentine carious lesion with *S. mutans* and *L. acidophilus* dual-species cariogenic biofilm. Med Oral Patol Oral Cir Bucal. (2013) 18:e824–31. 10.4317/medoral.18831 23722131 PMC3854072

[16] Zhao IS Gao SS Hiraishi N Burrow MF Duangthip D Mei ML. Mechanisms of silver diamine fluoride on arresting caries: a literature review. Int Dent J. (2018) 68:67–76. 10.1111/idj.12320 28542863 PMC9378923

[17] Paul B Sierra MA Xu F Crystal YO Li X Saxena D. Microbial population shift and metabolic characterization of silver diamine fluoride treatment failure on dental caries. PLoS ONE. (2021) 16:e0242396. 10.1371/journal.pone.0242396 33720954 PMC7959385

[18] Crystal YO Marghalani AA Ureles SD Wright JT Sulyanto R Divaris K. Use of silver diamine fluoride for dental caries management in children and adolescents, including those with special health care needs. Pediatr Dent. (2017) 39:135–45. 29070149

[19] Mira A. Oral microbiome studies: potential diagnostic and therapeutic implications. Adv Dent Res. (2018) 29:71–7. 10.1177/0022034517737024 29355422

[20] Tibshirani R. Regression shrinkage and selection via the lasso. J R Stat Soc Ser B. (1996) 58:267–88. 10.1111/j.2517-6161.1996.tb02080.x

[21] Hastie T Tibshirani R Friedman J. The Elements of Statistical Learning: Data Mining, Inference, and Prediction. New York2, NY: Springer (2016).

[22] Mei ML Lo ECM Chu CH. Arresting dentine caries with silver diamine fluoride: what's behind it? J Dent Res. (2018) 97:751–8. 10.1177/0022034518774783 29768975

[23] Nakano Y Suzuki N Kuwata F. Predicting oral malodour based on the microbiota in saliva samples using a deep learning approach. BMC Oral Health. (2018) 18:128. 10.1186/s12903-018-0591-6 30064419 PMC6069980

[24] Pietropaoli D Monaco A D'Aiuto F Muñoz Aguilera E Ortu E Giannoni M. Active gingival inflammation is linked to hypertension. J Hypertens. (2020) 38:2018–27. 10.1097/HJH.0000000000002514 32890278

[25] Chu CS Lee NP Adeoye J Thompson P Choi S-W. Machine learning and treatment outcome prediction for oral cancer. J Oral Pathol Med. (2020) 49:977–85. 10.1111/jop.13089 32740951

[26] Babji S Manickavasagam P Chen Y-H Jeyavelu N Jose NV Praharaj I. Immune predictors of oral poliovirus vaccine immunogenicity among infants in South India. NPJ Vaccines. (2020) 5:27. 10.1038/s41541-020-0178-5 32218999 PMC7089977

[27] Abdalla-Aslan R Yeshua T Kabla D Leichter I Nadler C. An artificial intelligence system using machine-learning for automatic detection and classification of dental restorations in panoramic radiography. Oral Surg Oral Med Oral Pathol Oral Radiol. (2020) 130:593–602. 10.1016/j.oooo.2020.05.012 32646672

[28] Seneviratne CJ Balan P Suriyanarayanan T Lakshmanan M Lee DY Rho M. Oral microbiome-systemic link studies: perspectives on current limitations and future artificial intelligence-based approaches. Crit Rev Microbiol. (2020) 46:288–99. 10.1080/1040841X.2020.1766414 32434436

[29] Ruff RR Niederman R. Silver diamine fluoride versus therapeutic sealants for the arrest and prevention of dental caries in low-income minority children: study protocol for a cluster randomized controlled trial. Trials. (2018) 19:523. 10.1186/s13063-018-2891-1 30257696 PMC6158895

[30] Huang SS Ruff RR Niederman R. An economic evaluation of a comprehensive school-based caries prevention program. JDR Clin Trans Res. (2019) 4:378–87. 10.1177/2380084419837587 31009589 PMC6749793

[31] Simón-Soro A Belda-Ferre P Cabrera-Rubio R Alcaraz LD Mira A. A tissue-dependent hypothesis of dental caries. Caries Res. (2013) 47:591–600. 10.1159/000351663 24080530

[32] Teng F Yang F Huang S Bo C Xu ZZ Amir A. Prediction of early childhood caries via spatial-temporal variations of oral microbiota. Cell Host Microbe. (2015) 18:296–306. 10.1016/j.chom.2015.08.005 26355216

[33] Zhang L Sun T Zhu P Sun Z Li S Li F. Quantitative analysis of salivary oral bacteria associated with severe early childhood caries and construction of caries assessment model. Sci Rep. (2020) 10:1–8. 10.1038/s41598-020-63222-1 32286402 PMC7156402

[34] Kanasi E Ayilavarapu S Jones J. Clonal analysis of the microbiota of severe early childhood caries. Caries Res. (2010) 44:485–97. 10.1159/000320158 20861633 PMC2975730

[35] Aas JA Paster BJ Stokes LN Olsen I Dewhirst FE. Bacteria of dental caries in primary and permanent teeth in children and young adults. J Clin Microbiol. (2008) 46:1407–17. 10.1128/JCM.01410-07 18216213 PMC2292933

[36] Sakamoto M Huang Y Umeda M Ishikawa I Benno Y. Prevotella multiformis sp. nov., isolated from human subgingival plaque. Int J Syst Evol Microbiol. (2005) 55:815–9. 10.1099/ijs.0.63451-0 15774668

[37] Long SS Prober CG Fischer M. Principles and Practice of Pediatric Infectious Diseases, Amsterdam:Elsevier (2017).

[38] Kataoka H Taniguchi M Fukamachi H Arimoto T Morisaki H Kuwata H. Rothia dentocariosainduces TNF-alpha production in a TLR2-dependent manner. Pathog Dis. (2014) 71:65–8. 10.1111/2049-632X.12115 24265267

[39] Eribe ERK Olsen I. Leptotrichia species in human infections II. J Oral Microbiol. (2017) 9:1368848. 10.1080/20002297.2017.1368848 29081911 PMC5646626

